# (2*E*)-1-(4-Chloro­phen­yl)-3-(4-nitro­phen­yl)prop-2-en-1-one

**DOI:** 10.1107/S1600536813010854

**Published:** 2013-04-27

**Authors:** T. S. Yamuna, H. S. Yathirajan, Jerry P. Jasinski, Amanda C. Keeley, B. Narayana, B. K. Sarojini

**Affiliations:** a Department of Studies in Chemistry, University of Mysore, Manasagangotri, Mysore 570 006, India; b Department of Chemistry, Keene State College, 229 Main Street, Keene, NH 03435-2001, USA; c Department of Studies in Chemistry, Mangalore University, Mangalagangotri 574 199, India; d Department of Chemistry, P.A. College of Engineering, Nadupadavu, Mangalore 574 153, India

## Abstract

In the title compound, C_15_H_10_ClNO_3_, a substituted chalcone, the dihedral angle between the benzene rings is 5.1 (7)°. The nitro group makes a dihedral angle of 12.5 (3)° with the benzene ring to which it is attached. In the crystal, weak C—H⋯O inter­actions link the mol­ecules into a one-dimensional array along [010]. The crystal studied was an inversion twin, with a refined ratio for the twin components of 0.6060 (9):0.3939 (1).

## Related literature

For the biochemical activity of chalcones, see: Dimmock *et al.* (1999 ▶). For different chalcone derivatives, see: Samshuddin *et al.* (2010 ▶); Fun *et al.* (2010*a* ▶,*b* ▶); Jasinski *et al.* (2010*a* ▶,*b* ▶); Baktır *et al.* (2011*a* ▶,*b* ▶). For related structures, see: Jing (2009 ▶); Jasinski *et al.* (2008 ▶, 2010*a* ▶,*b* ▶); Fun *et al.* (2011 ▶); Sarojini *et al.* (2007 ▶); Ma (2007 ▶).
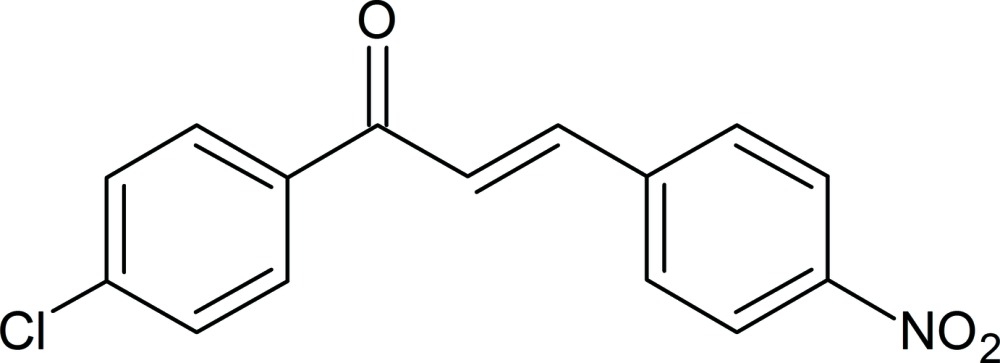


## Experimental

### 

#### Crystal data

C_15_H_10_ClNO_3_
*M* *_r_* = 287.69
Orthorhombic, 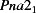

*a* = 42.9266 (17) Å
*b* = 5.9741 (3) Å
*c* = 5.0680 (2) Å
*V* = 1299.68 (10) Å^3^
*Z* = 4
Cu *K*α radiation
μ = 2.67 mm^−1^
*T* = 173 K
0.42 × 0.08 × 0.04 mm

#### Data collection

Agilent Xcalibur (Eos, Gemini) diffractometer
Absorption correction: multi-scan (*CrysAlis PRO* and *CrysAlis RED*; Agilent, 2012 ▶) *T*_min_ = 0.803, *T*_max_ = 1.000
12814 measured reflections
2538 independent reflections
2481 reflections with *I* > 2σ(*I*)
*R*_int_ = 0.037

#### Refinement

*R*[*F* ^2^ > 2σ(*F* ^2^)] = 0.048
*wR*(*F* ^2^) = 0.100
*S* = 1.14
2538 reflections
182 parameters
1 restraint
H-atom parameters constrained
Δρ_max_ = 0.22 e Å^−3^
Δρ_min_ = −0.39 e Å^−3^

### 

Data collection: *CrysAlis PRO* (Agilent, 2012 ▶); cell refinement: *CrysAlis PRO*; data reduction: *CrysAlis PRO*; program(s) used to solve structure: *SHELXS97* (Sheldrick, 2008 ▶); program(s) used to refine structure: *SHELXL2012* (Sheldrick, 2008 ▶); molecular graphics: *OLEX2* (Dolomanov *et al.*, 2009 ▶); software used to prepare material for publication: *OLEX2*.

## Supplementary Material

Crystal structure: contains datablock(s) global, I. DOI: 10.1107/S1600536813010854/fj2627sup1.cif

Structure factors: contains datablock(s) I. DOI: 10.1107/S1600536813010854/fj2627Isup2.hkl

Supplementary material file. DOI: 10.1107/S1600536813010854/fj2627Isup3.cml

Additional supplementary materials:  crystallographic information; 3D view; checkCIF report

## Figures and Tables

**Table 1:** Hydrogen-bond geometry (Å, °)

*D*—H⋯*A*	*D*—H	H⋯*A*	*D*⋯*A*	*D*—H⋯*A*
C12—H12⋯O2^i^	0.93	2.69	3.304 (4)	125
C14—H14⋯O1^ii^	0.93	2.53	3.219 (4)	131

Symmetry codes: (i) 
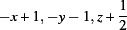
; (ii) 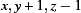.

**Fig. 1.**
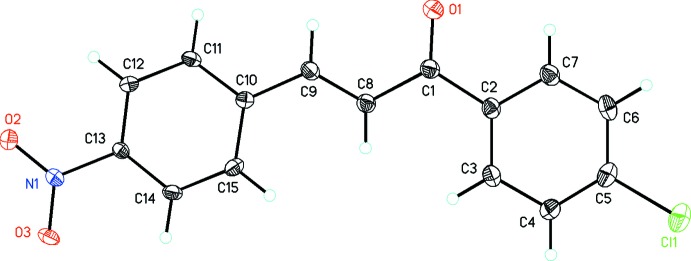
Molecular structure of the title compound showing the atom labeling scheme and 30% probability displacement ellipsoids.

**Fig. 2.**
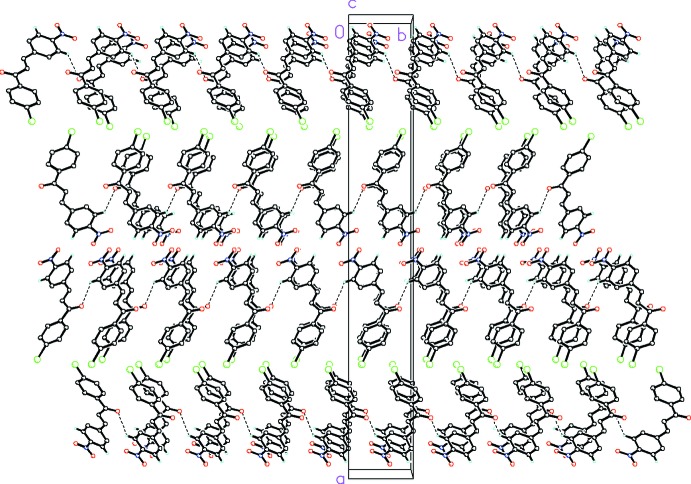
Packing diagram of the title compound viewed along the c axis. Dashed lines indicate weak C—H···O intermolecular interactions. H atoms not involved as weak intermolecular interactions have been deleted for clarity.

**Fig. 3.**
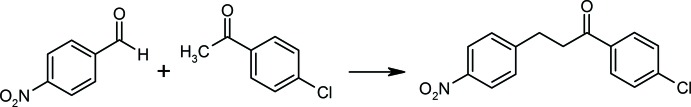
Reaction scheme.

## supplementary crystallographic information

### Comment

Chalcones can be easily obtained from the Claisen-Schmidt reaction of aromatic aldehydes and aromatic ketones. Chalcones have been reported to possess many useful properties including anti-inflammatory, antimicrobial, antifungal, antioxidant, cytotoxic, antitumour and anticancer activities (Dimmock *et al.* 1999). The basic skeleton of chalcones which possess α,β-unsaturated carbonyl group is useful synthone for the synthesis of various biodynamic cyclic derivatives such as pyrazoline, benzodiazepine and cyclohexenone derivatives (Samshuddin *et al.*, 2010; Fun *et al.*, 2010*a*,*b*; Jasinski *et al.*, 2010*a*; Baktır *et al.*, 2011a,b). The crystal structures of some of chalcones containing nitro group, viz., (E)-1-(4-nitrophenyl)-3-phenylprop-2-en-1-one (Jing, 2009), (2E)-3-(4-methylphenyl)-1-(3-nitrophenyl)prop-2-en-1-one (Jasinski *et al.*, 2008), (2E)-3-(2-chlorophenyl)-1-(3-nitrophenyl)prop-2-en-1-one (Sarojini *et al.*, 2007); (2E)-1-(2,5-dimethoxyphenyl)-3-(3-nitrophenyl)prop-2-en-1-one (Fun *et al.*, 2011) and (E)-3-(4-methoxyphenyl)-1-(3-nitrophenyl)prop-2-en-1-one (Ma, 2007) have been reported. In continuation of our work on synthesis of chalcones (Jasinski *et al.*, 2010*b*) we report here in the crystal structure of the title compound C_15_H_10_ClNO_3_, (I).

In (I), the dihedral angle between the mean planes of the 4-chlorophenyl and 4-nitrophenyl rings is 5.1 (7)° (Fig. 1). The nitro group makes a dihedral angle of 12.5 (3)° with the plane of the benzene to which it is bonded. In the crystal, weak C—H···N intermolecular interactions are observed and contribute to packing stability (Fig. 2). The crystal studied was an inversion twin, the refined ratio of the twin components being 0.6060 (9):0.3939 (1).

### Experimental

To a mixture of 4-nitrobenzaldehyde (1.51 g, 0.01 mol) and 4-chloroacetophenone (1.54 g, 0.01 mol) in ethanol (50 ml), 10 ml of 10% sodium hydroxide solution was added and stirred at 278-283 K for 3 hours (Fig. 3). The precipitate formed was collected by filtration and purified by recrystallization from ethanol. Single crystals were grown from acetone by the slow evaporation method (m.p.: 413–418 K).

### Refinement

All of the H atoms were placed in their calculated positions and then refined using the riding model with Atom—H lengths of 0.93Å (CH). Isotropic displacement parameters for these atoms were set to 1.2 (CH) times *U*_eq_ of the parent atom.

### Crystal data

**Table:**

C_15_H_10_ClNO_3_	*D*_x_ = 1.470 Mg m^−^^3^
*M**_r_* = 287.69	Cu *K*α radiation, λ = 1.5418 Å
Orthorhombic, *P**n**a*2_1_	Cell parameters from 5168 reflections
*a* = 42.9266 (17) Å	θ = 3.3–32.2°
*b* = 5.9741 (3) Å	µ = 2.67 mm^−^^1^
*c* = 5.0680 (2) Å	*T* = 173 K
*V* = 1299.68 (10) Å^3^	Rod, colorless
*Z* = 4	0.42 × 0.08 × 0.04 mm
*F*(000) = 592	

### Data collection

**Table:**

Agilent Xcalibur (Eos, Gemini) diffractometer	2481 reflections with *I* > 2σ(*I*)
Detector resolution: 16.1500 pixels mm^-1^	*R*_int_ = 0.037
ω scans	θ_max_ = 89.4°, θ_min_ = 7.5°
Absorption correction: multi-scan (*CrysAlis PRO* and *CrysAlis RED*; Agilent, 2012)	*h* = −55→55
*T*_min_ = 0.803, *T*_max_ = 1.000	*k* = −7→7
12814 measured reflections	*l* = −4→6
2538 independent reflections	

### Refinement

**Table:**

Refinement on *F*^2^	Hydrogen site location: inferred from neighbouring sites
Least-squares matrix: full	H-atom parameters constrained
*R*[*F*^2^ > 2σ(*F*^2^)] = 0.048	*w* = 1/[σ^2^(*F*_o_^2^) + (0.0202*P*)^2^ + 1.4764*P*] where *P* = (*F*_o_^2^ + 2*F*_c_^2^)/3
*wR*(*F*^2^) = 0.100	(Δ/σ)_max_ < 0.001
*S* = 1.14	Δρ_max_ = 0.22 e Å^−^^3^
2538 reflections	Δρ_min_ = −0.39 e Å^−^^3^
182 parameters	Absolute structure: Refined as an inversion twin.
1 restraint	Flack parameter: 0.39 (3)

### Special details

**Table:**

Geometry. All esds (except the esd in the dihedral angle between two l.s. planes) are estimated using the full covariance matrix. The cell esds are taken into account individually in the estimation of esds in distances, angles and torsion angles; correlations between esds in cell parameters are only used when they are defined by crystal symmetry. An approximate (isotropic) treatment of cell esds is used for estimating esds involving l.s. planes.
Refinement. Refined as a 2-component inversion twin.

### Fractional atomic coordinates and isotropic or equivalent isotropic displacement parameters (Å^2^)

**Table:**

	*x*	*y*	*z*	*U*_iso_*/*U*_eq_	
Cl1	0.25813 (2)	−0.1905 (2)	1.5999 (3)	0.0500 (3)	
O1	0.36902 (7)	−0.7676 (5)	0.9840 (8)	0.0475 (9)	
O2	0.49287 (6)	−0.1656 (4)	−0.2914 (6)	0.0318 (6)	
O3	0.46280 (6)	0.1223 (4)	−0.2523 (6)	0.0325 (6)	
N1	0.47115 (6)	−0.0656 (5)	−0.1880 (6)	0.0233 (6)	
C1	0.36318 (8)	−0.5698 (6)	0.9597 (9)	0.0275 (8)	
C2	0.33781 (7)	−0.4654 (6)	1.1220 (9)	0.0263 (7)	
C3	0.32630 (8)	−0.2524 (6)	1.0729 (9)	0.0326 (9)	
H3	0.3351	−0.1653	0.9406	0.039*	
C4	0.30164 (9)	−0.1685 (7)	1.2203 (9)	0.0359 (9)	
H4	0.2936	−0.0270	1.1850	0.043*	
C5	0.28918 (8)	−0.2975 (7)	1.4195 (9)	0.0317 (9)	
C6	0.30051 (9)	−0.5094 (7)	1.4746 (9)	0.0340 (9)	
H6	0.2918	−0.5950	1.6091	0.041*	
C7	0.32480 (8)	−0.5907 (7)	1.3268 (9)	0.0311 (9)	
H7	0.3328	−0.7320	1.3639	0.037*	
C8	0.38006 (8)	−0.4296 (6)	0.7654 (8)	0.0263 (8)	
H8	0.3743	−0.2807	0.7436	0.032*	
C9	0.40321 (7)	−0.5122 (6)	0.6217 (9)	0.0258 (7)	
H9	0.4088	−0.6602	0.6527	0.031*	
C10	0.42078 (7)	−0.3904 (5)	0.4177 (7)	0.0204 (7)	
C11	0.44691 (8)	−0.4917 (5)	0.3052 (8)	0.0238 (7)	
H11	0.4531	−0.6323	0.3636	0.029*	
C12	0.46373 (7)	−0.3858 (5)	0.1074 (8)	0.0220 (7)	
H12	0.4811	−0.4538	0.0320	0.026*	
C13	0.45406 (7)	−0.1771 (5)	0.0260 (7)	0.0188 (7)	
C14	0.42835 (7)	−0.0721 (5)	0.1327 (8)	0.0242 (7)	
H14	0.4223	0.0688	0.0737	0.029*	
C15	0.41184 (8)	−0.1789 (6)	0.3268 (8)	0.0261 (8)	
H15	0.3944	−0.1097	0.3993	0.031*	

### Atomic displacement parameters (Å^2^)

**Table:**

	*U*^11^	*U*^22^	*U*^33^	*U*^12^	*U*^13^	*U*^23^
Cl1	0.0414 (5)	0.0599 (7)	0.0487 (6)	0.0036 (5)	0.0136 (5)	−0.0095 (7)
O1	0.0412 (15)	0.0303 (14)	0.071 (2)	0.0065 (12)	0.0231 (16)	0.0156 (16)
O2	0.0357 (14)	0.0297 (13)	0.0301 (15)	0.0025 (10)	0.0093 (12)	0.0015 (12)
O3	0.0381 (14)	0.0251 (13)	0.0343 (16)	0.0044 (10)	0.0002 (12)	0.0136 (13)
N1	0.0264 (14)	0.0223 (13)	0.0213 (16)	−0.0014 (11)	−0.0021 (12)	0.0037 (12)
C1	0.0201 (16)	0.0260 (17)	0.036 (2)	−0.0017 (13)	−0.0007 (15)	0.0055 (17)
C2	0.0204 (14)	0.0298 (18)	0.0286 (19)	−0.0037 (12)	−0.0013 (16)	0.0020 (18)
C3	0.0343 (18)	0.032 (2)	0.031 (2)	−0.0026 (15)	0.0078 (18)	0.0044 (19)
C4	0.0338 (19)	0.0298 (18)	0.044 (3)	0.0037 (15)	0.0088 (19)	−0.0001 (19)
C5	0.0233 (16)	0.039 (2)	0.033 (2)	−0.0030 (15)	−0.0001 (16)	−0.0133 (18)
C6	0.0293 (18)	0.043 (2)	0.029 (2)	−0.0116 (17)	0.0002 (17)	0.005 (2)
C7	0.0252 (17)	0.0332 (19)	0.035 (2)	−0.0038 (14)	−0.0031 (16)	0.0070 (18)
C8	0.0228 (16)	0.0266 (17)	0.029 (2)	−0.0004 (13)	0.0001 (15)	0.0043 (17)
C9	0.0240 (15)	0.0263 (16)	0.0271 (19)	−0.0012 (13)	−0.0001 (16)	0.0059 (18)
C10	0.0188 (14)	0.0201 (15)	0.0223 (18)	−0.0006 (12)	−0.0033 (13)	0.0003 (14)
C11	0.0261 (16)	0.0184 (15)	0.027 (2)	0.0011 (12)	−0.0018 (14)	0.0059 (16)
C12	0.0220 (14)	0.0189 (14)	0.0252 (18)	0.0030 (12)	−0.0021 (15)	−0.0006 (16)
C13	0.0206 (14)	0.0187 (14)	0.0172 (17)	−0.0016 (12)	−0.0021 (12)	0.0017 (13)
C14	0.0256 (15)	0.0188 (15)	0.028 (2)	0.0034 (12)	−0.0015 (15)	0.0053 (16)
C15	0.0228 (16)	0.0236 (16)	0.032 (2)	0.0053 (13)	0.0019 (15)	−0.0025 (17)

### Geometric parameters (Å, º)

**Table:**

Cl1—C5	1.738 (4)	C7—H7	0.9300
O1—C1	1.214 (4)	C8—C9	1.327 (5)
O2—N1	1.225 (4)	C8—H8	0.9300
O3—N1	1.222 (4)	C9—C10	1.472 (5)
N1—C13	1.469 (4)	C9—H9	0.9300
C1—C8	1.482 (5)	C10—C11	1.397 (5)
C1—C2	1.501 (5)	C10—C15	1.398 (5)
C2—C3	1.388 (5)	C11—C12	1.388 (5)
C2—C7	1.396 (5)	C11—H11	0.9300
C3—C4	1.389 (5)	C12—C13	1.377 (4)
C3—H3	0.9300	C12—H12	0.9300
C4—C5	1.378 (6)	C13—C14	1.380 (4)
C4—H4	0.9300	C14—C15	1.370 (5)
C5—C6	1.384 (6)	C14—H14	0.9300
C6—C7	1.372 (6)	C15—H15	0.9300
C6—H6	0.9300		
			
O3—N1—O2	123.8 (3)	C9—C8—C1	121.4 (3)
O3—N1—C13	117.8 (3)	C9—C8—H8	119.3
O2—N1—C13	118.3 (3)	C1—C8—H8	119.3
O1—C1—C8	121.1 (4)	C8—C9—C10	125.8 (3)
O1—C1—C2	119.9 (3)	C8—C9—H9	117.1
C8—C1—C2	119.0 (3)	C10—C9—H9	117.1
C3—C2—C7	118.9 (4)	C11—C10—C15	118.5 (3)
C3—C2—C1	122.7 (4)	C11—C10—C9	119.0 (3)
C7—C2—C1	118.4 (3)	C15—C10—C9	122.5 (3)
C2—C3—C4	120.4 (4)	C12—C11—C10	121.0 (3)
C2—C3—H3	119.8	C12—C11—H11	119.5
C4—C3—H3	119.8	C10—C11—H11	119.5
C5—C4—C3	119.2 (4)	C13—C12—C11	118.2 (3)
C5—C4—H4	120.4	C13—C12—H12	120.9
C3—C4—H4	120.4	C11—C12—H12	120.9
C4—C5—C6	121.5 (4)	C12—C13—C14	122.4 (3)
C4—C5—Cl1	118.5 (3)	C12—C13—N1	118.8 (3)
C6—C5—Cl1	120.0 (3)	C14—C13—N1	118.9 (3)
C7—C6—C5	118.7 (4)	C15—C14—C13	118.9 (3)
C7—C6—H6	120.6	C15—C14—H14	120.5
C5—C6—H6	120.6	C13—C14—H14	120.5
C6—C7—C2	121.3 (4)	C14—C15—C10	121.0 (3)
C6—C7—H7	119.3	C14—C15—H15	119.5
C2—C7—H7	119.3	C10—C15—H15	119.5
			
O1—C1—C2—C3	−168.6 (4)	C8—C9—C10—C11	172.5 (4)
C8—C1—C2—C3	9.6 (6)	C8—C9—C10—C15	−9.2 (6)
O1—C1—C2—C7	9.9 (6)	C15—C10—C11—C12	0.0 (5)
C8—C1—C2—C7	−172.0 (3)	C9—C10—C11—C12	178.3 (3)
C7—C2—C3—C4	−1.9 (6)	C10—C11—C12—C13	0.4 (5)
C1—C2—C3—C4	176.5 (4)	C11—C12—C13—C14	−0.4 (5)
C2—C3—C4—C5	1.4 (7)	C11—C12—C13—N1	−178.4 (3)
C3—C4—C5—C6	−0.5 (6)	O3—N1—C13—C12	−177.9 (3)
C3—C4—C5—Cl1	−179.5 (3)	O2—N1—C13—C12	2.1 (5)
C4—C5—C6—C7	0.3 (6)	O3—N1—C13—C14	4.1 (5)
Cl1—C5—C6—C7	179.2 (3)	O2—N1—C13—C14	−176.0 (3)
C5—C6—C7—C2	−0.9 (6)	C12—C13—C14—C15	0.1 (5)
C3—C2—C7—C6	1.7 (6)	N1—C13—C14—C15	178.1 (3)
C1—C2—C7—C6	−176.8 (4)	C13—C14—C15—C10	0.3 (6)
O1—C1—C8—C9	−3.7 (6)	C11—C10—C15—C14	−0.3 (5)
C2—C1—C8—C9	178.3 (4)	C9—C10—C15—C14	−178.6 (4)
C1—C8—C9—C10	177.8 (3)		

### Hydrogen-bond geometry (Å, º)

**Table:**

*D*—H···*A*	*D*—H	H···*A*	*D*···*A*	*D*—H···*A*
C12—H12···O2^i^	0.93	2.69	3.304 (4)	125
C14—H14···O1^ii^	0.93	2.53	3.219 (4)	131

Symmetry codes: (i) −*x*+1, −*y*−1, *z*+1/2; (ii) *x*, *y*+1, *z*−1.
